# Rotator cuff repair with biological graft augmentation causes adverse tissue outcomes

**DOI:** 10.1080/17453674.2020.1793613

**Published:** 2020-07-21

**Authors:** Mustafa S Rashid, Richard D J Smith, Navraj Nagra, Kim Wheway, Bridget Watkins, Sarah Snelling, Stephanie G Dakin, Andrew J Carr

**Affiliations:** Nuffield Department of Orthopaedics, Rheumatology, and Musculoskeletal Sciences (NDORMS), Botnar Research Centre, Oxford, UK

## Abstract

Background and purpose — Biological patches can be used to augment rotator cuff tendon repair in an attempt to improve healing and reduce rates of re-rupture. However, little is known about the in vivo tissue response to these patches. We assessed native rotator cuff tissue response after surgical repair and augmentation with 2 commercially available extracellular matrix (ECM) patches.

Patients and methods — Patients underwent a rotator cuff repair augmented with either GraftJacket (Wright Medical), Permacol (Zimmer Biomet), or no patch (Control), applied using an onlay technique. A sample of supraspinatus tendon was collected intraoperatively and 4 weeks post-surgery, using ultrasound-guided biopsy. Histology and immunohistochemistry were performed on all samples.

Results — The Permacol group (n = 3) and GraftJacket group (n = 4) demonstrated some changes in native tendon ECM compared with the control group (n = 3). Significant disruption of the extracellular matrix of the repaired native supraspinatus, underlying both patches, was observed. The patches did not generally increase cellularity, foreign body giant cell count, or vascularity compared to the control group. 1 patient in the Permacol group had an adverse tissue immune response characterized by extensive infiltration of IRF5^+^, CD68^+^, and CD206^+^ cells, suggesting involvement of macrophages with a pro-inflammatory phenotype. No significant differences in protein expression of CD4, CD45, CD68, CD206, BMP7, IRF5, TGFß, and PDPN were observed among the groups.

Interpretation — Histological and immunohistochemical analysis of native tendon tissue after patch augmentation in rotator cuff repair raises some concerns about a lack of benefit and potential for harm from these materials.

Rotator cuff tendon tears occur in 1 in 3 people aged over 60 years (Tempelhof et al. 1999). Around 17,000 rotator cuff repairs are performed in the National Health Service (NHS) in the UK each year (Digital 2016). The incidence of rotator cuff repair is increasing in the UK and the USA (Colvin et al. 2012). Numerous observational studies have attempted to describe the healing rate following cuff repair (Russell et al. 2014, Shen et al. 2014, Yang et al. 2017). Despite the evolution in technique and implants, the overall healing rate is around 60% (Carr et al. 2017). This has led surgeons to develop innovative strategies that aim to augment tendon repair and improve healing rate.

One rotator cuff tendon repair augmentation strategy involves the application of a patch overlying the repair. These patches may be biological or synthetic. Biological patches are designed to become incorporated and vascularized by the native tendon, adding essential matrix proteins for healing (Zimmer 2006). Biological graft sources may be from the patient him/herself (e.g., fascia lata autograft), from cadaveric donors (e.g., dermal allograft), or from porcine tissues (e.g., dermal or small intestine submucosa, xenograft). These biological patches, sometimes called extracellular matrix (ECM) patches, are processed to remove donor cells, and sometimes chemically crosslinked, before sterilization for clinical use (Zimmer 2006, Group 2017). 2 popular biological patches, available for clinical use in rotator cuff repair, are GraftJacket (Wright Medical, Memphis, TE, USA) and Permacol (Zimmer Biomet, Warsaw, IN, USA).

GraftJacket Regenerative Tissue Matrix (RTM) (manufactured by LifeCell Corporation, Branchburg, NJ, USA) is a cadaveric human dermis graft that is not crosslinked and undergoes decellularization by a proprietary process (Group 2017). Permacol (manufactured by Tissue Science Laboratories PLC, Aldershot, UK) is a porcine dermis graft that is chemical crosslinked with 4,4'-Diisocyanato-methylenedicyclohexane (HMDI), and is decellularized by a proprietary process. Both GraftJacket and Permacol patches are marketed with some supporting information from in vitro and animal studies, showing cellular infiltration and neovascularization; however, the mechanisms underpinning these observations are unclear (McQuillan and Harper 2007, Xu et al. 2009, O’Brien et al. 2011, Xu et al. 2012).

The in vivo tissue response to xenograft and allograft tissue is important to consider in patch augmentation in humans. Patch augmentation in rotator cuff repair carries some additional risks. These include foreign-body reaction, sterile inflammatory response, transmission of undiagnosed malignancy, and infectious disease transmission (Hinsenkamp et al. 2012). We ascertained the tissue response of the native supraspinatus tendon to 2 biological patches at 4 weeks compared with a control (no patch), using histology and immunohistochemistry.

## Patients and methods

This study was conducted on patients undergoing rotator cuff repair. 2 groups of patients underwent rotator cuff repair and augmentation with 2 types of patches. These were GraftJacket (Wright Medical, Memphis, TE, USA) and Permacol (Zimmer Biomet, Warsaw, IN, USA). The control group received conventional rotator cuff repair without augmentation. The primary endpoint was an ultrasound-guided core biopsy sample of all patients in the 3 groups (GraftJacket, Permacol, or control), 4 weeks after surgery. The 4-week time point was chosen to represent early tissue response, and initial inflammatory response. Inclusion criteria included patients with a symptomatic, atraumatic, full-thickness tear involving the supraspinatus ± infraspinatus tendon(s), confirmed with ultrasound or magnetic resonance imaging (MRI). Patients who failed nonoperative treatment including physiotherapy, rest, analgesia, and/or corticosteroid injection(s) were included. Exclusion criteria included partial thickness tears, irreparable tears, acute traumatic tears, subscapularis tears, and patients who had not undergone a trial of conservative management. The primary outcome was native supraspinatus tendon tissue response, assessed by H&E staining, at 4 weeks post-surgery. Secondary outcomes included inflammatory response within the native supraspinatus tendon, as assessed by immunohistochemistry panel, at 4 weeks post-surgery.

Baseline measurements including Oxford Shoulder Score (OSS), Visual Analogue Score (VAS), and EuroQol 5D questionnaire (EQ-5D) were recorded to help define the patient cohort, but not for clinical outcome evaluation.

13 individuals were sequentially allocated into 3 groups, Group 1 (GraftJacket augmentation, 4 patients), Group 2 (Permacol augmentation, 4 patients), and Group 3 (control – standard repair without augmentation, 5 patients). 3 patients were excluded from the tissue analysis. The reasons for exclusion were: partial thickness tear (n = 1), irreparable massive tear (n = 1), and postoperative deep infection requiring arthroscopic washout and debridement (n = 1). The patient with the partial thickness tear was allocated to the Permacol group and the latter 2 patients were in the control group. Thus, Group 1 (GraftJacket augmentation) included 4 patients; Group 2 (Permacol augmentation) included 3 patients; and Group 3 (control group/no patch augmentation) included 3 patients. Demographics, baseline, and 4-week outcomes of patients are listed in Table 2. The patients were treated from March 2016 to March 2017.

**Table 2.** The 13 patients enrolled in the study including demographic data, patient-reported outcome scores (Oxford Shoulder Score, OSS, Euroqol-5D, EQ-5D, and Visual Analogue Scale, VAS) at baseline

			Baseline		
	Size of		OSS	VAS	EQ-5D
Age	tear (cm)	Patch	(0–48)	(0–10)	(0–100)
52	5	GraftJacket	27	6.9	65
49	6	GraftJacket	27	5.2	80
63	5	GraftJacket	30	5.7	95
55	4	GraftJacket	12	5.0	85
67	4	Permacol	7	10.	45
60	6	Permacol	28	4.7	96
34	3	Permacol	41	1.3	70
72	5	Control	15	8.1	75
68	3	Control	27	7.0	80
63	5	Control	18	7.3	90
55 ^a^	4	Control	31	4.5	75
55 ^a^	8	Irreparable tear	39	2.5	90
62 ^a^	PTT	N/A	32	3.0	15

PTT = partial thickness tear.

**^a^**Patients excluded for reasons stated.

Surgical procedures were performed under general anesthesia with regional blockade (interscalene block). At the time of surgery, prior to supraspinatus tendon repair, a sample of the torn free edge of the tendon was harvested for histology and immunohistochemistry. Subjects underwent a single-row rotator cuff repair with Versalok (Depuy Mitek, Warsaw, IN, USA) and Healix BR (Depuy Synthes, Warsaw, IN, USA) suture anchors via a mini-open, deltoid-splitting approach. After the repair was completed, patients in Groups 1 and 2 had either a GraftJacket or Permacol patch applied in an onlay technique over the repair and secured with 3-0 PDS sutures as per the manufacturers’ instructions.

**Figure 1.**
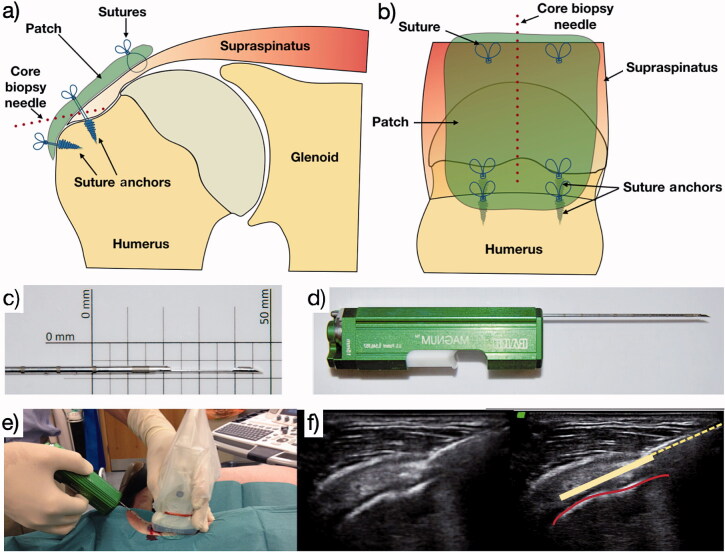
a and b: The needle site used at the 4-week postoperative biopsy; c and d: the core biopsy needle and automated firing device used; e and f: the technique for ultrasound-guided core biopsy. Red line denotes supraspinatus bony footprint. Dotted yellow line denotes core biopsy needle; solid yellow line denotes trajectory of core biopsy needle within supraspinatus tendon when deployed.

Postoperative rehabilitation was guided by a physical therapist and included 4 weeks of sling immobilization, followed by 2 weeks of passive range-of-motion exercises, and 6 weeks of active mobilization. At 4 weeks, an ultrasound (USS) guided biopsy under local anesthesia (2 mL 2% lignocaine) of the repaired supraspinatus tendon was performed with a Bard Magnum core biopsy system and a 16g tissue biopsy needle (Bard Peripheral Vascular, Inc, Tempe, AZ, USA) (Figure 1). This technique has been previously validated (Murphy et al. 2013).

All samples were fixed in 10% formalin, processed with a Leica ASP300S tissue processor (Leica Biosystems Nussloch GmbH, Nußloch, Germany), and embedded in paraffin wax. Using a rotary RM-2135 microtome (Leica Microsystems Ltd, Milton Keynes, UK), sections of 5–6 µm were cut and mounted on glass slides (Leica Microsystems Ltd, Milton Keynes, UK). These were baked at 60° C for 30 minutes, and then at 37° C for 60 minutes. For histology, H&E staining was performed using a Tissue Tek DRS automated stainer (Sakura Finetech Europe, Leiden, Netherlands). For immunohistochemistry (IHC), sections underwent high pH, heat-mediated, epitope retrieval using a PT Links machine (Dako Agilent Pathology Solutions, Santa Clara, CA, USA). Sections were then stained using a Dako Autostainer Link 48 machine (Dako Agilent Pathology Solutions, Santa Clara, CA, USA). Antibody staining was performed with the EnVision FLEX visualization system and an Autostainer Link 48 machine (Dako Agilent Pathology Solutions, Santa Clara, CA, USA) (Table 1).

**Table 1.** Primary antibodies used in immunohistochemistry (IHC), including details and rationale for use

Primary antibody	Source	Host	Clonality	Product code	Conc.	Rationale
CD4	Biorbyt	Rabbit	Polyclonal (IgG)	Orb182470	1:500	Glycoprotein co-receptor on surface of CD4^+^ T-helper cells
CD45	LSBio	Mouse	Monoclonal (IgG1)	LS-C187484	1:250	Leucocyte common antigen. Pan-leucocyte marker
CD68	Dako Agilent	Mouse	Monoclonal (IgG1)	IR609	1:1000	Transmembrane glycoprotein in monocyte lineage cells, e.g., monocytic phagocytes
CD206	Abcam	Rabbit	Polyclonal (IgG)	Ab64693	1:2000	Mannose receptor. Cell surface marker on macro- phages and immature dendritic cells
BMP7	Abcam	Mouse	Monoclonal (IgG1)	Ab54904	1:4000	BMP7 is part of TGFß superfamily. Counteracts TGFß1 in fibrosis, anti-fibrotic marker
IRF5	Proteintech	Rabbit	Polyclonal	10547-1-AP	1:300	Interferon Regulatory Factor 5. A transcription factor expressed by pro-inflammatory macrophages
TGFß	Abcam	Rabbit	Monoclonal (IgG)	Ab170874	1:150	Cytokine with pro-fibrotic effects
PDPN	Abcam	Mouse	Monoclonal (IgG1)	Ab10288	1:200	Stromal cell activation marker

Stained sections were imaged using a Zeiss AX10 inverted microscope with an Axiom HRc camera and Axiovision software (Zeiss, Cambridge, UK) at 40× and 100× magnification for H&E and IHC respectively. For histology sections, total cell count, foreign-body giant cell count (FBGC), and vascularity grading (0–3 scale) were performed on 6 random fields using ImageJ software (National Institutes of Health, Bethesda, MD, USA). This system has been previously validated (Rashid 2018). For IHC sections, all images were imported into CellProfiler software (Broad Institute, Cambridge, MA, USA) and a bespoke pipeline was applied to determine percentage of 3,3'-diaminobenzidine (DAB) staining per nuclei for each sample. For the purposes of analysis, histology and immunohistochemistry results were analyzed in groups as described earlier. Median change from preoperative to 4 weeks postoperative was used to compare across groups. Data were presented using GraphPad PRISM software (GraphPad software, Inc, La Jolla, CA, USA); however, numbers are too small to conduct meaningful statistical analysis.

### Ethics, funding, and potential conflicts of interest

This study was granted ethical approval by the National Healthcare Service (NHS) Research and Ethics Committee (REC) Ref: 15/SC/0697. This study was supported by a National Institute for Health Research (NIHR) Biomedical Research Unit (BRU) infrastructure grant. The authors declare no financial disclosures.

## Results

**Figure 2.**
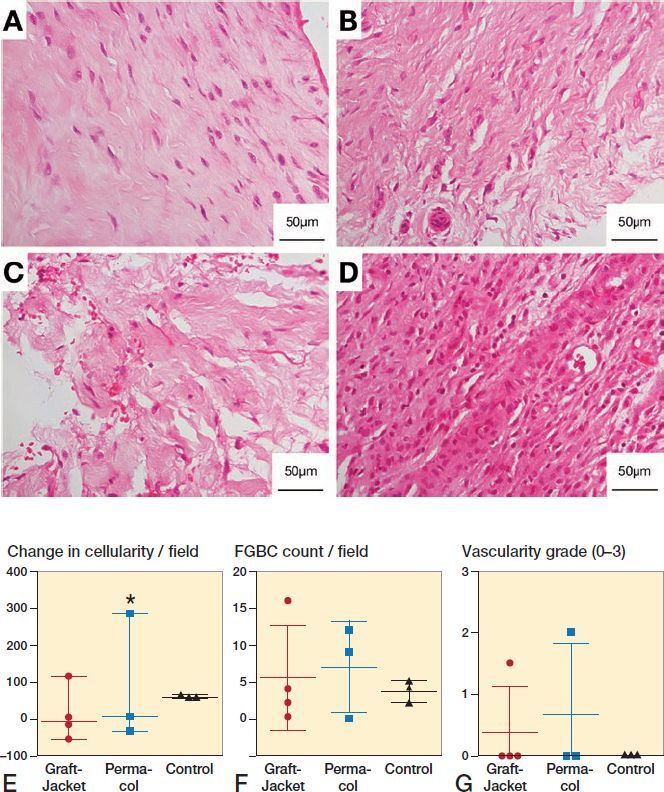
Representative histology showing tissue response to patch augmentation compared with control (no patch) group. A–D: Typical 4-week biopsy sections stained with hematoxylin and eosin (H&E) for control (A), GraftJacket (B), and Permacol (C) patch augmentation, showing increasing disruption of the tendon extracellular matrix (ECM). D: Abnormal tissue response from patient receiving Permacol showing dense infiltration of immune cells. E–G: Histology results comparing 3 groups (GraftJacket, Permacol, and Control) for change in cellularity (E), foreign-body giant cell (FBGC) count (F), and vascularity grade (G). * symbol denotes the patient receiving Permacol with grossly different tissue response.

Histology using H&E staining demonstrated significant disruption, on qualitative review, of the extracellular matrix (ECM) in the patch augmentation groups compared with the control group (Figure 2A–C). Specifically, sections demonstrated reduced crimp pattern, increased friability of the matrix, and lack of parallel oriented collagen fibers. Sections from the control group (conventional repair without patch augmentation) resembled a similar appearance to normal tendon. Permacol sections had more disruption of the ECM than GraftJacket sections on qualitative assessment. Results for total cell count, foreign-body giant cell count, and vascularity grading are presented in Figure 2. There was generally no significant difference between the groups; however, the tissue sections of one patient in Group 2 (Permacol patch) had a distinctly different histological appearance (Figure 2D). These sections showed markedly increased cellularity. Morphological features of cells suggest they are not tendon fibroblasts, as was seen in other patients’ tissue sections, but rather a dense infiltration of immune cells. Clinically, this patient complained of a painful arthrofibrosis 1-week post-surgery, which settled with analgesia from the general practitioner. The serum C-reactive protein level at 4 weeks was 10 mg/dL.

**Figure 3.**
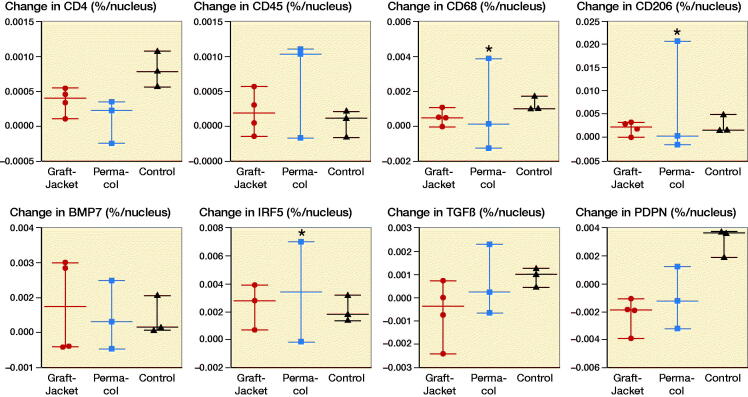
Immunohistochemistry (IHC) results showing change in immunopositive (DAB) staining (difference between preoperative and 4 week postoperative staining) per nucleus in 3 groups (GraftJacket, Permacol, and Control). A–G: Comparing change immunopositive staining per nuclei in 3 experimental groups for anti-CD4 (A), anti-CD45 (B), anti-CD68 (C), anti-CD206 (D), anti-BMP7 (E), anti-IRF5 (F), anti-TGFβ (G), and anti-PDPN (H). _*_ symbol denotes patient receiving a Permacol patch, with a grossly different tissue response, showing significantly higher immunopositive staining against CD68, CD206, and IRF5.

Immunohistochemistry staining revealed no differences between the 3 groups for the primary antibodies tested (Figure 3). However, the patient with the abnormal reaction in Group 2 demonstrated significantly increased immunopositive staining for CD68+, CD206+, and IRF5+ cells (marked by * in Figures 3C/D/F).

## Discussion

Patch augmentation is occasionally used to improve the healing rate in rotator cuff repair surgery. There are currently no prospective clinical studies that utilize post-implantation biopsies to investigate the human tissue response to these materials. This study is the first to characterize the early in vivo tissue response in humans undergoing rotator cuff repair with, and without, biological patch augmentation. We observed significant extracellular matrix disruption of the native supraspinatus tendon in response to both GraftJacket and Permacol patches compared with the control (no patch augmentation) group. The Permacol xenograft group demonstrated more ECM disruption of the native underlying supraspinatus tendon than the GraftJacket allograft group. At the early (4-week) time point, there was generally no increase in foreign-body giant cells or vascularity in both patch groups compared with the control (no patch) group.

1 patient who received the Permacol patch demonstrated significantly increased cellularity on rotator cuff tissue biopsy. This patient experienced a painful arthrofibrosis postoperatively. The tendon tissue sections from this patient were densely infiltrated with immune cells on histological evaluation. Immunohistochemistry did not reveal any significant differences between the groups; however, the sections from the aforementioned patient in group 2 showed a significant increase in CD68^+^/CD206^+^/IRF5^+^ cells, suggestive of an adverse immune response. Another patient, in the control group, developed a deep infection following uncomplicated rotator cuff repair, further highlighting the potential, albeit rare, risks of surgery.

The philosophy behind biological patch augmentation is that the native tendon tissue often lacks a capacity to heal to the bony footprint and that application of a collagen and elastin-rich decellularized graft, which can become integrated with native cells and blood vessels, would improve the healing rate (Zimmer 2006, Group 2017). Thus, the aim is to improve healing rate by improving the biological environment.

Several groups have investigated the response of tendon cells on GraftJacket and Permacol patches in the laboratory (Derwin et al. 2006, Fini et al. 2007, Smith et al. 2016). Human tendon-derived cells cultured on various synthetic and biological patches exhibited different morphology, indicating that physical cues may influence cell characteristics and protein expression (Smith et al. 2016). In particular, synthetic patches demonstrated healthy tenocyte morphology with extended lamellipodia, and increased collagen I:collagen III ratio. Cells cultured on biological patches such as GraftJacket and Permacol demonstrated atypical cell morphology (Smith et al. 2016).

Residual microDNA fragments have been observed in commercially available biological patches, raising concerns regarding a potential immune response (Derwin et al. 2006). Biological patches are derived from harvested cadaveric or animal tissue and processed under proprietary processes for implantation. Despite this, no 2 patches are the same, with variation in protein content and residual DNA content being observed. In our study, we did not biopsy each patch prior to implantation. Hence, we cannot comment on the cause of the sterile inflammatory reaction seen in the patient who received the Permacol patch.

In an infraspinatus repair canine model augmented with a human dermal graft, observed infiltration of the grafts occurred by 6 weeks on histology and chronic inflammation was noted. 2 of 10 failed repairs demonstrated increased inflammatory infiltrate that the authors concluded may represent rejection of the human dermal matrix graft (Adams et al. 2006). There is a paucity of clinical studies that have evaluated in vivo human tissue response to biological patches. Histological samples from an individual case report revealed extensive infiltration of noninflammatory host cells and blood vessels (Snyder et al. 2009). A case series of 4 patients undergoing bridging repair of large rotator cuff tears with Permacol showed very poor results (Soler et al. 2007). All 4 patients failed to improve. Fluid in the subdeltoid bursa was seen in all patients on MRI (1 aspirated to confirm sterile effusion). 2 of 4 patients went on to have a reverse total shoulder replacement. At the time of surgery, it was noted that histology demonstrated chronic inflammation with necrotic fibrous material (Soler et al. 2007).

Despite over 1 million implantations of GraftJacket (in a wide range of surgical applications) (Group 2017), and over 100,000 implantations for Permacol (mainly in genitourinary and hernia repair surgery) (Zimmer 2006), there are no high-quality, low risk of bias, clinical studies evaluating their clinical efficacy. There are several clinical studies investigating the application of GraftJacket or Permacol in rotator cuff repair augmentation, all with significant bias. Most studies are observational, all of which demonstrated some improvement in patient-reported outcome measures (Burkhead et al. 2007, Bond et al. 2008, Wong et al. 2010, Gupta et al. 2012, Kokkalis et al. 2014). Only 1 study included a comparator group, reported as a level II randomized controlled trial (Barber et al. 2012). This quasi-randomized controlled trial of patients undergoing rotator cuff repair for large posterosuperior rotator cuff tears included 42 patients, randomized to either conventional arthroscopic repair (n = 20), or arthroscopic repair plus augmentation of GraftJacket as an onlay (n = 22). They observed no adverse events related to the patches, although one patient developed bursitis postoperatively. This study suffers from several limitations and there are serious concerns for risk of bias in trial design, conduct, and reporting, as assessed by the Cochrane risk of bias tool domains (Higgins et al. 2011).

Our study has limitations that must be considered in light of the results. First, the numbers are small and our findings of no difference in the immunohistochemistry are, therefore, not conclusive. Additionally, the small numbers in each group precluded robust statistical analysis. This study does demonstrate that postoperative biopsy is possible and well tolerated by patients. Second, with any research involving core biopsies, despite best efforts to standardize there is some variance with the location of each biopsy. All ultrasound-guided biopsies were performed by an experienced shoulder ultrasonographer (AJC). We were limited only in commenting on the native tendon tissue response because not all biopsies included a sample of the patch. Given that the time point for these biopsies is early (4 weeks), we would not expect to see much cellular infiltration or neovascularization within the patch tissue at this time. Third, the tissue response was assessed at only 1 time point. We chose to focus on the early response in the belief that the early phases of healing are most important, and any acute inflammatory reaction would be observed at this time. We cannot comment on changes that may occur at earlier or later time points. Fourth, there are currently no specific and sensitive cell markers for identifying tendon fibroblasts exclusively. Most commonly used cell markers also stain other immune cell types. Hence, we chose to use total cellularity in the histology sections to identify differences between the groups. Finally, this study was not designed to demonstrate clinical efficacy of one patch over another. For this, a well-designed randomized controlled trial would answer the question of whether patch augmentation is superior to a control group (e.g., no patch augmentation). Both patches are licensed and used widely for rotator cuff repair augmentation, despite initially being developed for other, non-musculoskeletal applications. The human rotator cuff tendon enthesis is complex, and if patch augments are to make significant contributions towards improved healing, a more tailored approach of scaffolds specifically designed for this purpose may be beneficial.

## Conclusions

This is the first study to systematically examine the native tissue response to commercially available biological patches applied in rotator cuff augmentation in humans. Significant disruption of the native supraspinatus tendon ECM was observed in the GraftJacket and Permacol patch augmented groups, compared with the control (no patch) group. 1 patient in the Permacol group had an adverse tissue reaction characterized by extensive infiltration of pro-inflammatory CD68^+^/CD206^+^/IRF5^+^ cells. These finding raise concerns regarding the use of these patches in rotator cuff augmentation on the basis of early tissue response in vivo.
